# Circulating membrane aminophospholipids contribute to thrombotic risk in rheumatoid arthritis

**DOI:** 10.1016/j.jlr.2025.100842

**Published:** 2025-06-14

**Authors:** Daniela O. Costa, Majd B. Protty, Victoria J. Tyrrell, Ali A. Hajeyah, Bethan H. Morgan, Ben Mead, Martin Giera, Peter W. Collins, P. Vince Jenkins, Ernest Choy, Simon A. Jones, Valerie B. O’Donnell

**Affiliations:** 1 Division of Infection and Immunity, Systems Immunity Research Institute, School of Medicine, Cardiff University, Cardiff, UK; 2 School of Optometry and Vision Sciences, Cardiff University, Cardiff, UK; 3 Center for Proteomics and Metabolomics, Leiden University Medical Center, Leiden, Netherlands; 4 Hematology Department, University Hospital of Wales, Cardiff, UK

**Keywords:** rheumatoid arthritis, thrombosis, lipidomics, extracellular vesicles, aminophospholipids

## Abstract

Patients with rheumatoid arthritis (RA) are at elevated risk of thrombotic events, yet the underlying mechanisms remain unknown. The contribution of the procoagulant membrane surface provided by aminophospholipids (aPLs) in driving thrombotic risk in RA has not been investigated. Specifically, neither the type of aPL exposed on circulating blood cell membranes in patients is characterized nor is their ability to support thrombin generation is known. Here, lipidomics was used to characterize the external-facing and total levels of aPL molecular species in RA, specifically phosphatidylserine and phosphatidylethanolamine on extracellular vesicles (EVs), platelets, and white blood cells (WBCs). The ability of the cells and EVs to support thrombin generation from patients and healthy controls was compared using an in vitro prothrombinase assay. RA patient plasma had significantly higher levels of thrombin-antithrombin and d-dimers, indicating increased thrombotic activity in vivo. Higher EV and platelet counts were seen in RA, but WBC counts were not elevated. EVs from RA patients supported higher levels of thrombin generation compared with healthy controls, whereas for platelets and WBC, thrombin generation was similar for both groups. EVs from RA patients also showed elevated external-facing phosphatidylserine molecular species, with total aPL also increased. For platelets and WBC, total and external-facing aPL levels were similar. Thrombin-antithrombin (TAT) complexes significantly correlated with EV particle counts, indicating that their circulating numbers are directly related to coagulation in vivo. Overall, our data suggest that elevated plasma EV levels in RA are a major source of procoagulant membranes, contributing to thrombotic risk in RA.

## Graphical abstract

**Figure:**
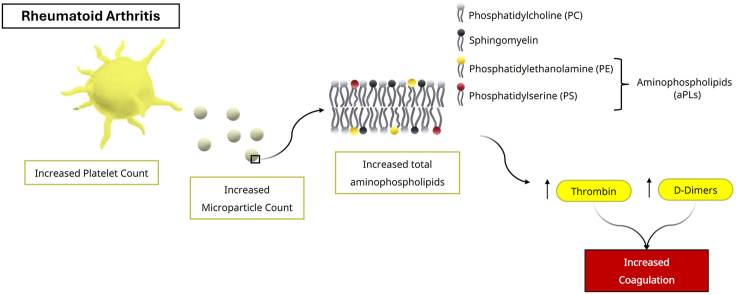

Rheumatoid arthritis (RA) is associated with an increased incidence of venous and arterial thrombosis (1, 2, 3). Several molecular markers of coagulation are known to be raised in RA plasma, including thrombin-antithrombin (TAT) complexes and d-dimers (4, 5, 6). An elevated immune response that drives chronic inflammation is often proposed to explain the increase in thrombosis, although the underlying mechanisms involved are currently unknown.

A primary contributor to coagulation is the aminophospholipid (aPL) membrane of platelets and circulating leukocytes, which forms on cell activation. In resting platelets or white blood cells (WBCs), the plasma membrane is asymmetrical with phosphatidylcholine and sphingomyelin being the most abundant phospholipids (PLs) in the outer leaflet, and aPLs such as phosphatidylserine (PS) and phosphatidylethanolamine (PE), mainly on the inner side (7). On activation, aPLs are externalized, where they support calcium-dependent binding and assembly of coagulation factors. Thus, PS is essential for coagulation, whereas PE boosts the activity of PS (8, 9, 10, 11, 12). While the role of this membrane in supporting normal hemostasis is well known, its contribution to elevated thrombotic risk in human disease is not fully understood. WBCs also drive coagulation through expressing tissue factor. This protein stimulates the formation of the extrinsic tenase complex along with factor VIIa and calcium, supporting thrombin generation (13).

Extracellular vesicles (EVs) are also considered key regulators of coagulation. These are derived from the plasma membrane of activated blood cells following membrane bending and budding (14). EVs are known to be a source of aPL for coagulation factor binding and activation, and they also contain tissue factor (15). Their role in driving thrombotic risk in RA is not known.

To date, the specific molecular species of PE and PS on the outside leaflet of membranes in RA have not been characterized. Increased immune cell activation, as evidenced by elevated annexin V binding to the surfaces of platelets and EVs, has been reported for patients with RA, and this has been suggested to represent increased PS externalization (16). However, annexin V is sensitive to PE externalization and furthermore does not report the types or amounts of PS molecular species present.

In this study, platelets, WBC, and plasma-derived EVs will be isolated from RA patients and healthy controls (HCs), and their total and external-facing PE and PS molecular species were characterized and quantified using LC-MS/MS. Their ability to drive thrombin generation using a prothrombinase assay will also be determined and compared with their circulating thrombotic activity.

## Materials and methods

### Human clinical samples

Human studies were performed under the project titled *Cardiff Regional Experimental Arthritis Treatment and Evaluation Centre* approved by the Ethics Committee for Wales (Reference no.: 12/WA/0045) and performed in accordance with the principles of the Declaration of Helsinki, with informed consent and with full ethical approval. RA patients were recruited for venous blood sampling during a routine clinic appointment. Patients had no history of venous and/or arterial thrombosis at the time of venipuncture. Gender-matched healthy volunteers were recruited, excluding volunteers with a history of arterial or venous thrombosis, recurrent fetal loss, cardiac disease, or any other chronic inflammatory diseases, such as diabetes and high cholesterol or any other diseases that may conflict with the study. The average age of HC was around 10 years lower, because of the unavailability of older subjects (Supplemental Table S1). Healthy subjects were requested to not take aspirin, nonsteroidal anti-inflammatory drugs, or any other medications for 14 days prior to blood donation. Recruitment ran from February 2020 to April 2022. Clinical characteristics of patients and controls are given in Supplemental Table S1.

### Washed platelet isolation from human blood

Human blood was collected using a 21-gauge butterfly needle and two 20 ml syringes. Each syringe contained 3.6 ml of acid-citrate-dextrose (2.5% [w/v] trisodium citrate, 1.5% [w/v] citric acid, and 100 mM glucose), with blood drawn until a final volume of 18 ml was obtained. The blood was centrifuged without brake (400 *g*, 10 min, 22°C). Platelet-rich plasma was isolated and recentrifuged without brake (1,400 *g*, 8 min, 22°C). Platelet-poor plasma (PPP) supernatant was transferred to Eppendorf tubes for EV isolation and coagulation assays. The platelet pellet was resuspended with 10 ml of acid-citrate-dextrose: Tyrode's buffer (145 mM NaCl, 12 mM NaHCO_3,_ 2.95 mM KCl, 1 mM MgCl_2_, 10 mM Hepes, and 5 mM glucose) (1:9 v/v), before centrifuging (1,400 *g* without brake, 8 min, 22°C). Platelets were resuspended in 1 ml Tyrode's buffer. Platelets were counted with a hemocytometer and resuspended at 2 × 10^8^ cells per ml in Tyrode's buffer. For lipid analysis, platelets (3 × 10^8^ cells in 1.5 ml) were incubated with/without 0.2 U/ml thrombin and 1 mM CaCl_2_ for 30 min at 37°C. Following this, lipids were immediately derivatized and extracted as outlined later. Platelets were alternatively used in prothrombinase assays, as outlined later. Partial clotting of blood led to two HC (platelets) being excluded from counting and lipid analysis.

### Coagulation markers

TAT complexes were quantified in PPP using a human TAT ELISA (ab108907; Abcam). Plasma was incubated with a biotinylated TAT complex-specific antibody before detection using a streptavidin-peroxidase conjugate. Chromogen substrate was added and left to react for 20 min before adding stop solution. Absorbance was immediately read on a microplate reader (CLARIOstar Plus; BMG Labtech) at 450 nm, and values were corrected for background by subtracting readings at 570 nm. All samples and standards were analyzed in duplicate. d-dimers were analyzed in plasma on a Werfen ACL TOP 750 using HemosIL® D-Dimer HS-500 latex agglutination reagent. A clinically raised d-dimer level was defined as >500 ng/ml. For a small number of samples, insufficient plasma remained for analysis following lipidomics. This impacted six HCs and two RA samples for TATs, nine HCs and four RA samples for d-dimer, as well as one HC and two RA samples for EV counts.

### EV isolation from human blood

PPP was centrifuged (17,000 *g*, 30 min). The top layer was discarded, and 750 μl of Tyrode's buffer was added. To generate a washed EV fraction, an additional centrifugation was performed (17,000 *g*, 30 min). The bottom 5% fraction was considered an EV-rich fraction and used for lipid extraction and prothrombinase assays, detailed later. This isolation method has been previously validated to be free of lipoprotein contamination, specifically of ApoB (17). EV count and size analysis were performed using the Nanoparticle Tracking Analysis instrument (ZetaView® TWIN, PMX-23; Particle Metrix, UK), after 20,000× dilution, using the following instrument parameters:

Laser wavelength: 488 nm, filter wavelength: scatter, sensitivity: 75, shutter: 100.

### WBC isolation from human blood

Human blood was collected using a 21-gauge butterfly needle and a 60 ml syringe. A total of 20 ml of blood was drawn into a syringe containing 4 ml HetaSep™ (Stem Cell Technologies, France) and 4 ml 2% citrate. After gentle inversion, the syringe was left in an upright position for 1 h to achieve gravity separation of the blood components. Subsequently, the top layer was removed and centrifuged (400 *g* without brake, 10 min, 4°C). The pellet was resuspended in Dulbecco's PBS (DPBS) (no calcium, no magnesium; Gibco™; Thermo Fisher Scientific)/0.4% citrate, followed by centrifugation (400 *g*, 10 min, 4°C). A 5 ml of 0.2% hypotonic NaCl was added to the pellet to lyse red blood cells, followed by a washing step with 50 ml of ice-cold DPBS/0.4% citrate. The WBCs were pelleted (400 *g*, 6 min, 4°C) and a second, where red blood cell lysis was performed. After pelleting the WBC (400 *g*, 5 min, 4°C), they were resuspended in 1 ml of Krebs buffer (0.1 M NaCl, 5 mM KCl, 47.7 mM Hepes, 1 mM NaH_2_PO_4_∙2H_2_O, 2 mM glucose, pH 7.4) and counted using a hemocytometer. Cells were then resuspended at 4 × 10^6^ WBC per ml in Krebs buffer. WBC (6 × 10^6^ cells in 1.5 ml) were incubated with/without 10 μM calcium ionophore A23187 and 1 mM CaCl_2_ for 30 min at 37°C, then lipids immediately derivatized and extracted, as described later. WBCs were separately used also for prothrombinase assays.

### Biotinylated standards

Biotinylated PL standards (DMPE-B, DMPS-B, SOPS-B, SAPS-B, SAPE-B, SpAPE-B, and DOPS-B) were generated as previously described (18). Briefly, lipids (1 mg) were dried under nitrogen (N_2_) and reconstituted in 330 μl of chloroform:methanol (2:1 v/v). To each lipid, EZ-Link™ NHS-Biotin (ThermoFisher) was added to provide a final concentration of 52 mM, followed by the addition of 3.3 μl of triethylamine (Sigma-Aldrich) and incubation for 30 min. The excess EZ-Link™ NHS-Biotin was pelleted by centrifugation (500 *g*, 5 min, 20°C), and the solvent fraction with the biotinylated lipids was transferred to a new vial. To increase yield, the pelleted sediment was washed with 330 μl of chloroform:methanol (2:1 v/v), vortexed, centrifuged, and solvent recovered, and combined with the previously isolated solvent fraction. The lipids were dried under N_2_, and biotinylated lipids resuspended in methanol before HPLC purification. Biotinylated standards were purified using HPLC on a Supelco Discovery C_18_ column with a 15 min gradient elution profile of 50% mobile phase B to 100% mobile phase B (A: 5 mM NH_4_CH_3_CO_2_ in water, B: 5 mM NH_4_CH_3_CO_2_ in methanol) and then held at 100% mobile phase B for 20 min. The biotinylated lipids were monitored at 205 nm and eluted from the column at approximately 20 min. They were collected, dried using a RapidVap Vacuum Dry Evaporation System (Labconco Corporation), weighed, and resuspended in methanol, before being capped under N_2_ atmosphere and stored at −80°C until use.

### Derivatization and extraction of lipids from platelets, WBCs, and EVs

External or total aPLs were detected using LC-MS/MS, following derivatization using biotin, which generates a mass shift of 226 a.m.u. (18). To measure external-facing aPL, 0.2 ml of cells/EVs were incubated with 86 μl of 11 mM EZ-Link™ Sulfo-NHS-biotin (ThermoFisher, UK) and dissolved in DPBS for 10 min at room temperature. The reaction was quenched using 72 μl of 250 mM l-lysine dissolved in DPBS, followed by a 10-min incubation. Finally, 42 μl of DPBS was added to bring the total volume to 400 μl. To measure total aPL, 0.2 ml of cells/EVs were incubated with 20 μl of 20 mM EZ-Link™ NHS-biotin, dissolved in DMSO, for 10 min. The final volume was made up to 0.4 ml by adding 180 μl DPBS. Following derivatization, 10 μl of internal standard mix (biotinylated DMPS and DMPE at 1 ng/ml) was added to all samples. PLs were extracted using the Bligh and Dyer method (19), as follows. Around 1.5 ml of chloroform:methanol (1:2 v/v) was added, and the sample was vortexed for 1 min. Next, 0.5 ml of CHCl_3_ was added, and the sample was again vortexed (1 min). Last, 0.5 ml of HPLC-grade water was added, before vortexing (1 min) and centrifuging (500 *g*, 5 min, 4°C). The lower phase was recovered using a glass Pasteur pipette and dried using a RapidVap Vacuum Dry Evaporation System before being resuspended in 100 μl of methanol and stored at −80°C prior to LC-MS/MS analysis.

### LC-MS/MS analysis of biotinylated aPL

Lipid extracts were separated using reversed-phase chromatography with an Ascentis C_18_ column (150 mm × 2.1 mm × 5 μm) (Sigma-Aldrich). An isocratic elution method was used with methanol (and 0.2% [w/v] NH_4_CH_3_CO_2_), at a flow rate of 400 μl/min for 25 min at 22°C. Lipids were analyzed in multiple reaction monitoring mode, on a 4000 Q-Trap (Sciex), and biotinylated multiple reaction monitoring transitions were monitored in negative ion mode, with a dwell time of 200 ms. For quantification, biotinylated standards were used to generate a standard curve. An equation for calculation was obtained using linear regression, and both the total and externalized concentrations of aPL were calculated. Limit of quantitation was determined as S/N >5, with at least six data points across a peak. A small number of samples were excluded because of identified errors in sample processing. This impacted four HCs for total aPLs, two HCs and four RAs (EVs only) for externalized aPLs, two HCs (EVs only) and three RAs.

### Prothrombinase assay

Thrombin generation was determined using a chromogenic assay adapted from previously described prothrombinase assays (20, 21). Twenty microliter platelets (4 × 10^6^ cells), WBC (8 × 10^4^ cells), or EVs (equivalent to 6 ml of plasma) were incubated with 20 μl coagulation factors (1 mM factor II, 50 nM factor Xa, 5 mM CaCl_2_ [Enzyme Laboratories, UK], 15 nM factor Va [Haematologic; Cambridge Bioscience]). After 5 min, the reaction was quenched using 7 mM EDTA. For the standard curve, a serial dilution of factor IIa, ranging from 3.125 to 400 nM, was used. Chromogenic substrate (S2238, 2.8 mM; Enzyme Research) was added to each well. Absorbance was read at 405 nm for 15 min on a microplate reader (CLARIOstar Plus). The area under the curve of the kinetic reaction was used for quantification. For prothrombinase of three RAs (EV, platelet, and WBC) and one RA (EVs only), samples were lost during analysis because of plate reader malfunction, whereas one EV sample (RA) could not be processed as insufficient blood was collected in the clinic.

### Statistical analysis

Statistical analysis was performed using GraphPad Prism 9 (GraphPad Software, Inc). The imputation method of missing values used half of the lowest detected concentration for each sample. Normality was determined using the Shapiro-Wilk test. The Kruskal-Wallis test was applied to nonparametric datasets. The statistical differences between conditions were calculated using Dunn's multiple comparisons test. Where data were normally distributed, a one-way ANOVA and Tukey's multiple comparison test were performed. Unless otherwise stated, data were displayed as boxplots, with median, and whiskers representing interquartile range. Heatmaps and hierarchical clustering were generated using the pheatmap package in R. Heatmaps show log_10_ mean lipid amounts (ng), normalized to cell count or volume (ml), allowing row-wise and column-wise comparison. Lipid levels were represented by a color gradient ranging from blue (very low levels or absent) to red (high levels). Outliers were not excluded from any analysis.

## Results

### d-dimers and TAT complexes are increased in RA

**Figure 1:**
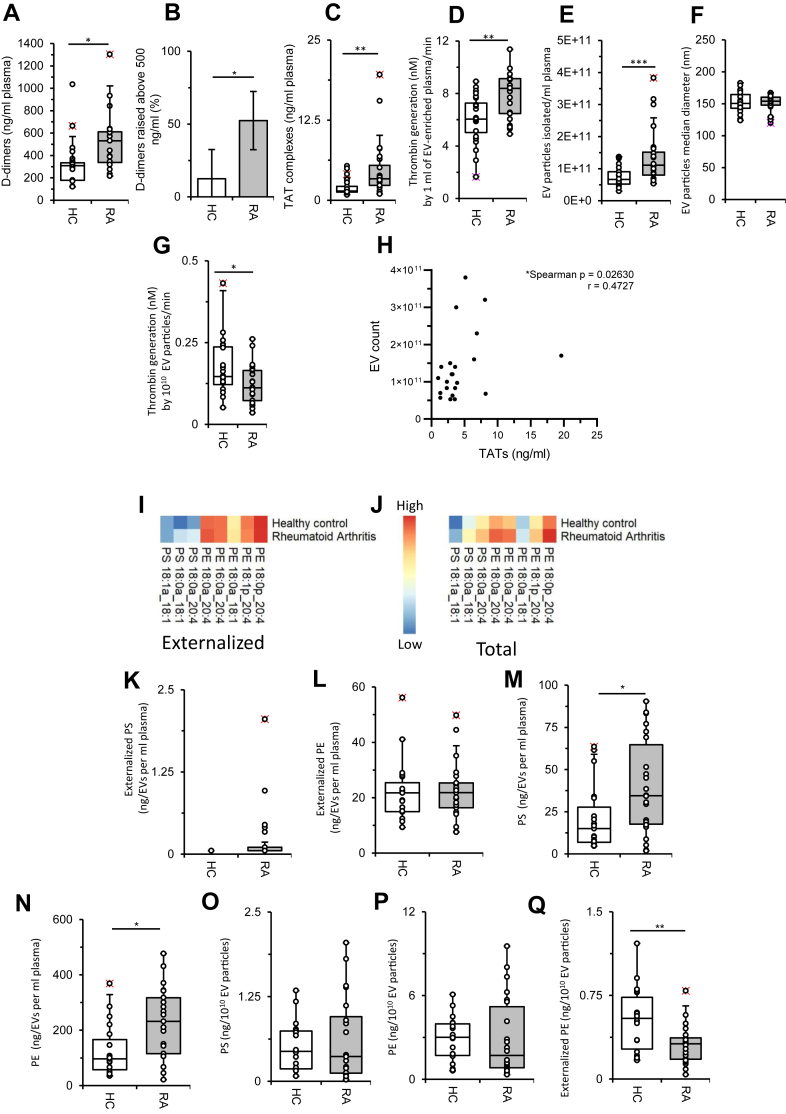
RA patients show higher coagulation markers and EV counts in plasma, and their EV can support increased in vitro thrombin generation. A and B: d-dimer levels are raised in RA patients. d-dimer levels were measured as described in Materials and methods section, using ELISA in plasma from RA patients (n = 21) and HC (n = 16), showing absolute levels (ng/ml) and frequency of raised levels (%) above clinical cutoff of 500 ng/ml shown. C: TAT complexes are increased in plasma from RA patients (22). TAT complexes were measured using ELISA in plasma from RA patients (n = 25) and HC (n = 19). D: EVs from RA patients generate more thrombin. The ability of EVs in 6 ml plasma to support coagulation reactions was assessed using the prothrombinase assay, as described in Materials and methods section, for HC (n = 24) and RA patients (n = 22). E and F RA patients have increased plasma EV counts but no increase in diameter. EV particles were counted, from HC (n = 24) and RA (n = 24) plasma, as described in Materials and methods section. G: Thrombin generation is increased in RA plasma because of increased EV levels. Prothrombinase assay, using 10^10^ EV particles, was applied to plasma from HC (n = 24) and RA (n = 21). Data were analyzed using Mann-Whitney test (∗*P* < 0.05, ∗∗*P* < 0.001, ∗∗∗*P* < 0.001). H: EV particle counts correlate with thrombin generation and TAT levels in RA. Spearman correlation was performed comparing EV particle counts with TAT complexes (n = 22) in RA plasma. I: Heatmap for external-facing PE and PS levels in RA and HC plasma. EV particles were isolated followed by biotinylation of externalized lipids, as described in Materials and methods section. Lipids were extracted from plasma from HC (n = 19) and RA patients (n = 21) as described in Materials and methods section and analyzed by LC-MS/MS. Externalized aPLs in EV are shown in a heatmap (log10, ng/ml). J: Total aPLs are increased in EVs from RA. Total (internal + external) individual aPLs in EVs were analyzed using LC-MS/MS and shown in a heatmap (log10, ng/ml). K and L: Externalized PS was barely detected in EVs from RA plasma and undetectable in HC, whereas external-facing PE was similar in both groups. Externalized PS and PE levels were determined (ng/ml) for HC (n = 19) and RA (n = 21), using LC-MS/MS as described in Materials and methods section. M and N: Total PS and PE levels are increased in EVs in RA. Total (external and internal) aPLs were quantified as described in Materials and methods section or HC (n = 19) and RA (n = 22) (ng/ml of plasma). O–Q*:* Externalized PE is decreased in EVs from RA patients following normalization of EV count. Total aPL concentrations were adjusted to EV counts (10^10^ EV particles), for HC (n = 18) and RA (n = 21), along with externalized PE for HC (n = 19) and RA patients (n = 20) (ng/10^10^ EV particles). Data were analyzed using the Mann-Whitney test (∗*P* < 0.05, ∗∗*P* < 0.01, and ∗∗∗*P* < 0.001).

Indices of coagulation were determined in RA cohort plasma. d-dimers were significantly increased in RA with 52.3% of patients exhibiting levels higher than the clinical cutoff of 500 ng/ml (Fig. 1A, B). TAT complexes were also significantly increased in RA (Fig. 1C) (22). These results confirm that patients with RA show elevated circulating indices of coagulation activity.

### EVs are increased in RA plasma and promote elevated thrombin generation ex vivo

Next, the ability of EV membranes to support thrombin generation was determined using a prothrombinase assay containing factors (factor II, factor Xa, and factor Va) and CaCl_2_, with thrombin (factor IIa) activity measured using a chromogenic substrate. EV fractions isolated directly from ∼6 ml of RA or HC plasma were first tested. Here, significantly higher thrombin generation was observed for RA-derived EV, compared with plasma from HC (Fig. 1D).

Next, plasma EVs were counted and found to be increased by about 50% in plasma from RA patients, in line with previous observations (Fig. 1E) (16). No difference in median EV diameter was found (Fig. 1F), confirming that these particles are consistent with microvesicles (23). The higher count most likely explains the increased ability of RA-derived EVs to stimulate thrombin generation, since we had not corrected for EV count before assessing thrombin generation. To test this, we normalized thrombin generation to individual EV counts and found that the trend reversed, with somewhat less per unit of EV (Fig. 1G). Evidencing the link between EVs and prothrombotic activity in vivo, a significant positive correlation was found between plasma TAT complexes and EV counts in RA plasma (Fig. 1H). This suggested that the higher EV levels in RA contribute to the increased thrombin generation observed both in vivo and ex vivo. Overall, these data indicate that RA plasma contains more EVs, supporting higher thrombin generation; however, individual EVs from RA patients appeared to be slightly less thrombogenic on a per-count basis.

### Total EV aPL levels are elevated in RA because of their higher plasma count

Next, the aPL membrane of EVs was characterized using LC-MS/MS. Two molecular species of PS (PS 18:0a_18:1, PS 18:0a_20:4) were detected on the outside of a very small number of RA patient samples, but in none of the HC plasmas (Fig. 1I, K), with external levels below the limit of detection of our assay. In the case of external-facing PE, several species (PE 18:0a_20:4, PE 16:0a_20:4, PE 18:0a_18:1, PE 18:1p_20:4, PE 18:0p_20:4) were detected, with the externalization pattern similar for RA and HC (Fig. 1I, L. Supplemental Fig. S1A). Next, when comparing total levels of PS (external and internal facing), it was seen that EVs from RA plasma contained significantly higher levels for most molecular species (Fig. 1J, M; Supplemental Fig. S1B). Similarly, total (external and internal facing) PE was significantly higher for EVs from RA (Fig. 1J, N; Supplemental Fig. S1C). Since EV counts were also significantly higher in patients with RA (Fig. 1E), we next considered whether PS levels or PE levels measured in patient EV isolates simply reflect the differences in EV numbers. Confirming this, once corrected to 10^10^ EV, the estimated number of particles per milliliter blood (24), total (internal and external facing) PE and PS levels became consistent for HC and RA groups (Fig. 1O, P). In contrast, external-facing PE became significantly lower for RA (Fig. 1Q). For external-facing PS, it was not possible to compare since external PS was below the limit of detection for the HC plasma-derived EVs. These data indicate that while external PS is detectable only on a few RA plasma-derived EV samples, external-facing PE levels are unchanged as a response to disease. Conversely, total EV-derived aPL levels are higher in RA plasma for both PS and PE, because of the higher particle numbers per ml. After normalizing for EV count, the proportion of external-facing PE was lower in RA patient particles. This may explain the reduced thrombin generation seen (Fig. 1G) for EVs from RA plasma, following their normalization to particle number. These data confirm that the increased total aPL in RA plasma is most likely a direct function of higher circulating EV numbers.

### RA platelets generate similar thrombin levels to HC; however, in vivo circulating counts are elevated in RA

**Figure 2:**
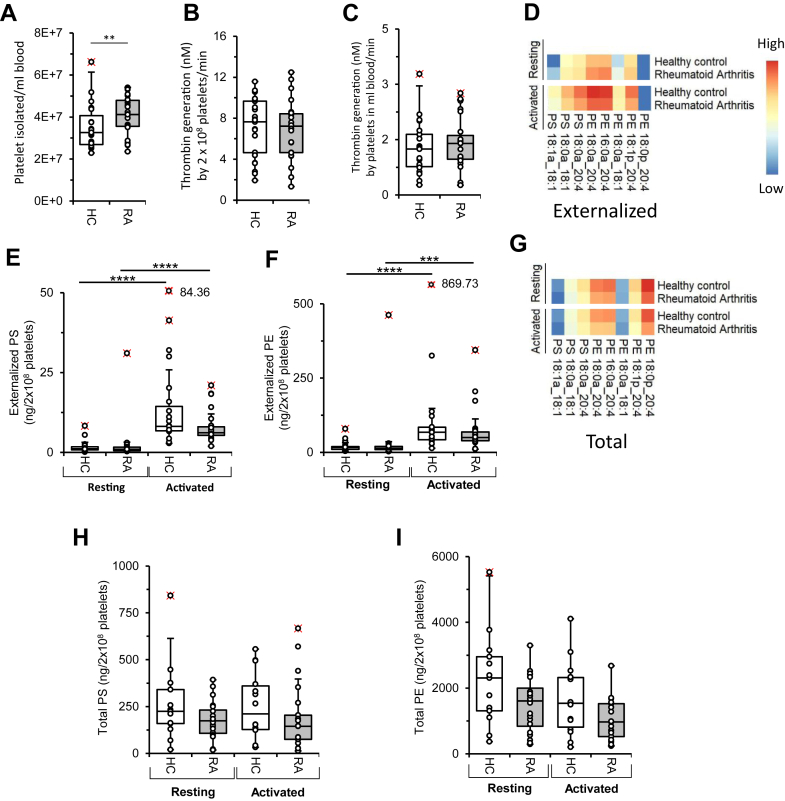
Platelet counts in RA are elevated, whereas PE externalization and thrombin generation are similar. A: Platelet count is increased in RA patients.^22^ Platelets were counted as described in Materials and methods section, HC (n = 23) and RA (n = 26). Data were analyzed using the Mann-Whitney test (∗∗*P* < 0.01). B and C: Thrombin generation by platelets is similar between RA and HC. Around 2 × 10^8^ platelets from HC (n = 25) and RA patients (n = 23) were assessed using the prothrombinase assay and expressed either on a per platelet basis or per blood platelet count from HC (n = 23) and RA patients (n = 23). Data were analyzed using Student's *t*-test. D: Externalized aPL profile is similar for RA and HC platelets. Lipids were extracted from resting platelets or following thrombin activation (0.2 U/ml). Externalized lipids were analyzed as described in Materials and methods section using LC-MS/MS. Platelet externalized aPL species, either resting or thrombin activated, from HC (n = 19) and RA patients (n = 22 and n = 23, respectively), were analyzed using LC-MS/MS and shown in heatmaps (log10, ng/2 × 10^8^ platelets). E and F: Externalized PS and PE levels are similar in resting or activated platelets from RA patients and HC. The sum of externalized PS in resting or activated platelets, along with externalized PE, was calculated, in HC (n = 19 for both) and RA patients (n = 22 and n = 23, respectively) (ng/2 × 10^8^ platelets). Data were analyzed using Kruskal-Wallis test and Dunn's multiple comparisons test (∗∗∗∗*P* < 0.0001). G: Platelets from RA patients contain less total aPLs. Total individual aPLs species in platelets, either resting or thrombin activated, from HC (n = 13 and n = 14) and RA patients (n = 20 and n = 21, respectively), were analyzed using LC-MS/MS, and are shown in heatmaps (log10, ng/2 × 10^8^ platelets). H and I: Resting platelets from RA patients have less total PE. The sum of total analyzed PS and PE was determined for both resting and thrombin-activated platelets, from HC (n = 13 and n = 14, respectively) and RA patients (n = 20 and n = 21, respectively), (ng/2 × 10^8^ platelets). Data were analyzed using the Kruskal-Wallis test and Dunn's multiple comparisons test.

Next, aPL and thrombin generation were determined in RA and HC patient platelets. Platelet counts were significantly increased in RA blood (Fig. 2A) (22). The ability of washed platelets to stimulate thrombin generation was measured using the same prothrombinase assay as for EVs, containing factors (factor II, factor Xa, and factor Va) and CaCl_2_, with thrombin (factor IIa) activity measured using a chromogenic substrate. In contrast to EVs, platelets will activate during this assay, externalizing aPLs to support the binding and activation of factors, and stimulating thrombin generated by the assay system. Here, no difference in thrombin production was observed when comparing a defined number of platelets from RA patients, with those from HC (Fig. 2B). Since RA patients display thrombocytosis, next we normalized thrombin generation to platelet count. Again, no difference was seen between the patient group and HC (Fig. 2C). This suggests that the elevated platelet count in RA may not directly contribute to higher thrombin generation. However, we recognize that the in vitro assay may not fully replicate the complexities associated with systemic chronic inflammation in RA patients.

### Total and externalized aPL levels in platelets show a trend to reduce in RA, which disappears after adjusting for cell counts

Next, PS and PE were analyzed in platelets from RA patients and HC. As previously shown, both PS and PE were externalized following thrombin activation of HC platelets (Fig. 2D) (12). Platelets from RA patients showed a nonsignificant reduction in PE or PS externalization compared with HC (Fig. 2D–F, Supplemental Fig. S2). This disappeared when aPL externalization was adjusted for platelet count (Supplemental Fig. S3). Total PS and PE (internal and external facing) was also analyzed and found to be unchanged before or after activation, despite a trend to be decreased in RA patient samples (Fig. 2H–I, Supplemental Fig. S4). Nevertheless, a significant decrease in total PE was found upon thrombin activation of platelets, especially in RA patients, indicating phospholipase hydrolysis (Supplemental Fig. S5).

### Circulating WBC counts and thrombin generation are similar in RA and HC

**Figure 3:**
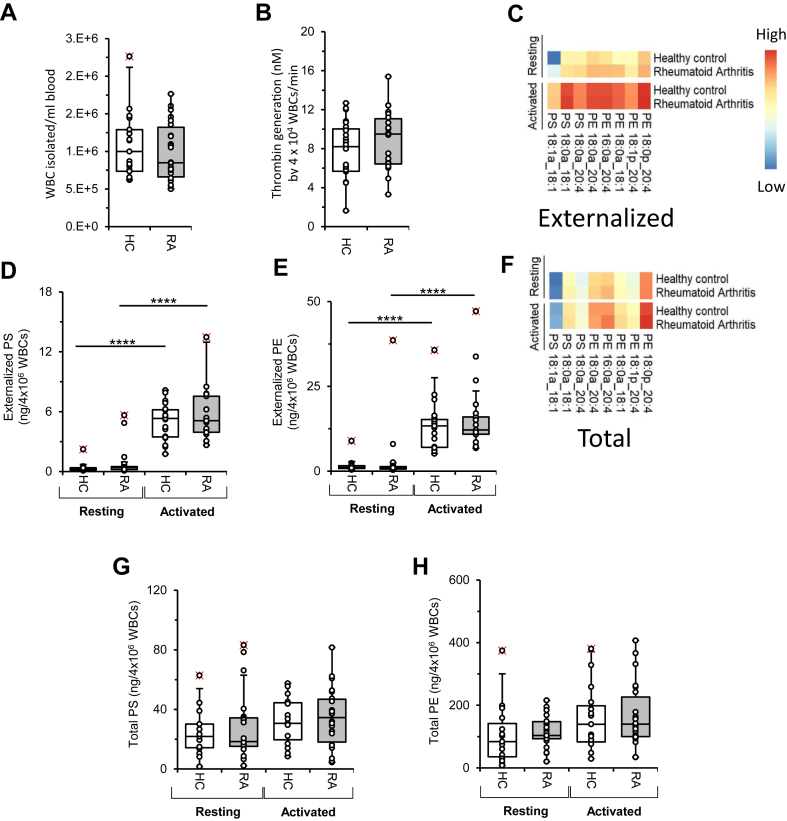
WBC counts are similar in RA and HC, whereas WBC from RA support similar thrombin generation and contain similar levels of total and external aPL to HC. A: WBC counts were similar between RA patients and HC (22). WBCs were counted in samples from HC (n = 25) and RA patients (n = 26), as described in Materials and methods section. B: Thrombin generation is similar for WBC from RA patients and HC. WBCs were tested for their ability to support prothrombinase activity, as described in Materials and methods section from HC (n = 24) RA (n = 22). Data were analyzed using Student's *t*-test. C: Externalization of aPLs in resting or activated WBCs from RA patients and HC is similar. Lipids were extracted from resting and ionophore-activated (10 μM) WBC, followed by analysis of externalized lipids as described in Materials and methods section using LC-MS/MS. Externalized individual aPL species in WBC, either resting or activated, from HC (n = 20 and n = 19, respectively) and RA patients (n = 23 and n = 21, respectively), were analyzed using LC-MS/MS, and shown in heatmaps (log10, ng/4 × 10^6^ WBC). D and E: Levels of external-facing PS and PE in WBC are similar for RA patients and HC. The sum of externalized PS and PE in resting and ionophore-activated WBC, in both HC (n = 20 and n = 19, respectively) and RA patients (n = 23 and n = 21, respectively), was calculated (ng 4 × 10^6^ WBs). Data were analyzed using the Kruskal-Wallis test and Dunn's multiple comparisons test (∗∗∗∗*P* < 0.0001). F: Total amount of aPLs in resting or activated WBC from RA patients and HC is similar. Total amounts of individual aPL species in WBC, either resting or activated, from HC (n = 15 for both) and RA patients (n = 21 for resting and n = 22 for activated), were analyzed using LC-MS/MS, and are shown in heatmaps (log10, ng/4 × 10^6^ WBC). G and H: Total PS and PE levels are similar for resting or activated WBC in RA patients and HC. The sum of total PS, along with total PE, was determined as described in Materials and methods section, in WBC, either resting or following ionophore activation, HC (n = 15 for both) and RA patients (n = 21 for resting and n = 22 for activated) (ng/4 × 10^6^ WBC). Data were analyzed using the Kruskal-Wallis test and Dunn's multiple comparisons test.

Since WBCs can also provide a procoagulant surface, we next tested for thrombin generation in cells isolated from RA or HC blood, using the same assay as used above for platelets and EVs. There were no differences in WBC counts or thrombin generation found between the groups (Fig. 3A,B) (22). This indicates that WBCs are unlikely to contribute to the higher thrombotic risk in RA via stimulating thrombin generation. To examine this further, we next characterized aPL levels and externalization in WBC.

### Total and external aPLs in WBCs are similar in RA and HC

Ionophore activation of WBCs leads to significant externalization of PS and PE (17). Here, levels of external-facing aPL were not different between RA and HC WBCs, before or after ionophore activation, indicating that this membrane compartment was not altered in disease (Fig. 3C–E, Supplemental Fig. S6). Total (external and internal combined) WBC levels of PS and PE were also measured and, unexpectedly, were found to be slightly increased following ionophore activation (Fig. 3F–H, Supplemental Fig. S7), although no differences were observed between RA and HC (Supplemental Fig. S8). This contrasts with the decrease in total PE following activation found in platelets (Supplemental Fig. S5). These data are in line with our WBC thrombin generation data showing no difference between RA and HC and considering the WBC counts are not changed in disease, these cells are unlikely to be contributing to increased thrombotic risk.

## Discussion

RA is a complex immune-mediated inflammatory disease that includes comorbidities associated with prothrombotic and cardiovascular complications. Furthermore, new therapeutic agents, namely target Janus-activated kinase inhibitors, more specifically tofacitinib in higher doses, have been associated with increased risk of blood clots (https://www.ema.europa.eu/en/medicines/human/referrals/xeljanz). Our results show that circulating d-dimers and TATs are elevated in RA patients suggesting they should be monitored as indices of coagulation risk in routine clinical assessments.

To determine the contribution of the procoagulant aPL membrane to this phenomenon, we used lipidomics and thrombin generation assays to characterize the membranes of EVs, platelets, and WBCs in RA. EV counts were elevated in RA blood, and based on their ability to support prothrombinase, as shown in this study, they appear to be a major source of circulating membranes contributing to elevated thrombotic risk in RA. On the other hand, platelets and WBCs did not clearly contribute to elevated in vitro thrombin generation in our study.

This is the first description of aPL molecular species in EVs, platelets, and WBCs from RA patients. Up to now, published studies rely on flow cytometry and fluorescent probes, such as annexin V, for the nonspecific and nonquantitative detection of total aPL (16, 25, 26, 27). Through the use of an MS assay, where primary amines on aPL headgroups are derivatized using a biotin tag, PS and PE were not only quantified, but external facing and total amounts both were determined (18). The specific aPL composition of EVs is not well characterized, having only been recently described in cardiovascular disease (17). The molecular species of PS in RA microparticles were PS 18:0a_18:1 and PS 18:0a_20:4, and increased levels in disease are consistent with previous reports of increased surface binding of annexin V to microparticles isolated from RA plasma (16, 26, 27). Molecular species of PE were more abundant than PS, with PE 18:0a_20:4, PE 18:0p_20:4, PE 18:0a_18:1, PE 16:0a_20:4, and PE 18:1p_20:4 detected. The aPL composition of EVs seen here for RA patients is similar to those described by Protty *et al.* (17) for EVs in atherosclerotic cardiovascular disease patients.

Activated blood cells and platelets generate various types of EVs from their membranes, including microparticles, exosomes, and apoptotic bodies. Increased EV counts have been previously reported in RA blood and synovial fluid, with over 90% shown to be platelet derived (28). The average size of the EVs in our study (≈150 nm), along with their procoagulant ability, confirms them to be microparticles, rather than exosomes, which do not bind and activate prothrombin (23). Furthermore, we previously excluded lipoprotein contamination of our isolated EVs (17). Microparticles derived from platelets have been shown to have increased procoagulant ability compared with activated platelets based on surface area (29). Furthermore, platelet-derived microparticles augment fibrin and platelet deposition in damaged arterial walls, promoting arteriosclerosis (30). These are likely the same particles that are elevated herein in RA. The increased TAT levels detected in RA plasma may be at least partially explained by these higher circulating EV counts. In support, a similar correlation was previously described for systemic lupus, another autoimmune disease with increased thrombotic risk (31).

Our data show no significant difference in WBC numbers or aPL composition between RA patients and HC, suggesting a lack of a role for leukocytes in the prothrombotic risk found in these patients. In contrast, platelet number was significantly increased in RA, in agreement with the published literature describing thrombocytosis in these patients (32). Intriguingly, total PE was significantly decreased in resting platelets from RA patients compared with HC. This suggests that platelets from RA patients may be less prothrombotic on a per-cell basis, although because of higher circulating counts, this is negated. Microparticles are considered to be generated through exocytic budding of the membrane of the parent cell in response to activation (33). Therefore, the increased aPL exposure, which occurs on activation, will also result in an increase of these lipids in microvesicles and consequently might lead to their depletion from the parent cell. Another potential contributor are nonsteroidal anti-inflammatory drugs, including aspirin and ibuprofen, which were administered to 38% of RA patients in this cohort. Since these can reduce secondary platelet activation, a reduction in aPL externalization might be dependent on these drugs, although this is not proven.

Overall, these data characterize for the first time the aPL molecular species and their amounts in circulating blood cells and EVs in RA. The study demonstrates that EVs, in particular microparticles, are likely to be a primary contributor to membrane-dependent coagulation, adding a new mechanism underpinning elevated thrombotic risk in this disease. The aPL composition of EVs, or their number in circulation, could be regarded as a future therapeutic target for the reduction of thrombotic risk in RA.

## Data availability

This article’s dataset is available in the Research Data Repository of Cardiff University at https://doi.org/10.17035/cardiff.29355968.v1.

## Supplemental data

This article contains supplemental data.

## Conflict of interest

E. C. has received research grants and honoraria from Abbvie, Alfasigma, Bio-Cancer, Biocon, Biogen, Chugai Pharma, Eli Lilly, Fresenius Kai, Galapagos, Gedeon Richter, Gilead, Inmedix, Janssen, Pfizer, Sanofi, UCB, and Viatris. S. A. J. has received funding support from Hoffman-La Roche, GlaxoSmithKline, Ferring Pharmaceuticals, Meastag Therapeutics, and NovImmune. S. A. J. has acted as an advisory consultant for Roche, Chugai Pharmaceuticals, NovImmune SA, Genentech, Sanofi Regeneron, Johnson & Johnson, Janssen Pharmaceuticals, Eleven Biotherapeutics, and Mab Design. V. O. D. is a consultant for Metasight.
