# Supplementary material for: Circulating membrane aminophospholipids contribute to thrombotic risk in rheumatoid arthritis
Source: J Lipid Res. 2025 Jun 14;66(7):100842. doi: 10.1016/j.jlr.2025.100842 (PMC12273563; doi:10.1016/j.jlr.2025.100842)
Supplement: Supplemental Figures [file mmc1.pdf]

SUPPLEMENTAL INFORMATION:

Circulating membrane aminophospholipids contribute to thrombotic risk in rheumatoid arthritis.

Daniela O Costa[1], Majd B Protty[1], Victoria J Tyrrell[1], Ali A Hajeyah[1], Beth Morgan[1], Ben Mead[4,] Martin Giera[3], Peter W Collins[1,2], P Vince Jenkins[2], Ernest Choy[1], Simon A Jones[1], Valerie B O'Donnell[1]

[1] Systems Immunity Research Institute, Division of Infection and Immunity, School of Medicine, Cardiff University, CF14 4XN, UK

[2] Hematology Department, University Hospital of Wales, CF14 4XN Cardiff, UK

[3] Center for Proteomics and Metabolomics, Leiden University Medical Center, Leiden, Netherlands

[4] School of Optometry and Vision Sciences, Cardiff University, UK

Variable	Healthy control (HC) [n=25]	Rheumatoid Arthritis (RA) [n=26]	p
**Age, Mean ± SD**	51.69 ± 8.74	61.08 ± 16.51	0.0224 (b)
**Female sex**	21 (84%)	23 (88%)	0.6514 (a)
**DAS28, Mean ± SD**	–	2.68 ± 1.45	–
**Disease duration (years), Mean ± SD**	–	7.58 ± 8.43	–
**Rheumatoid Factor (+)**	–	60%	–
**Anti-CCP (+)**	–	64%	–
**Erythrocyte sedimentation rate (mm/hour ± SD)**	-	14.12 ± 12.9	-
**CRP (mg/L), Mean ± SD**	-	4.8 ± 7.37	-
**Aspirin use**	0	8%	–
**NSAIDs use**	0	30%	–
**Smoker**	0		–
**Osteoarthritis**	0	23%	–
**Hypertension**	0	19%	–
**Diabetes**	0	0	–
**Hypothyroidism**	0	12%	–
**Statin use**	0	0	–

Supplementary Table 1: Baseline clinical characteristics of recruited volunteers in the clinical cohort

(CCP: Cyclic Citrullinated Peptide, CRP: c-reactive protein, DAS: disease score activity, p-value tests: Fisher exact for categorical (a) or Mann-Whitney test for continuous variables (b)).

Supplementary Figures

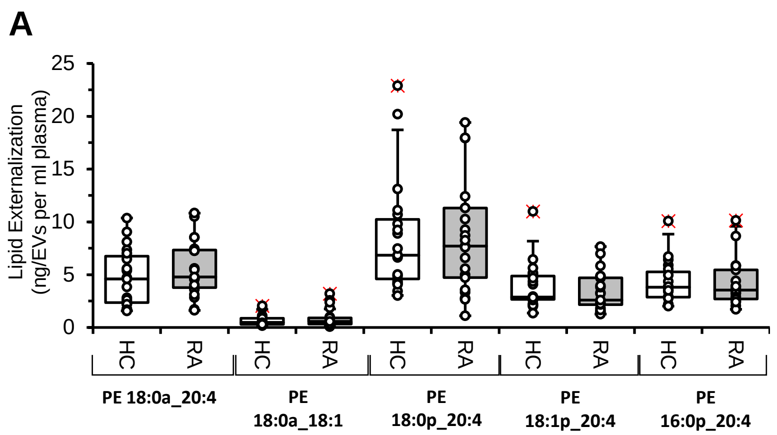

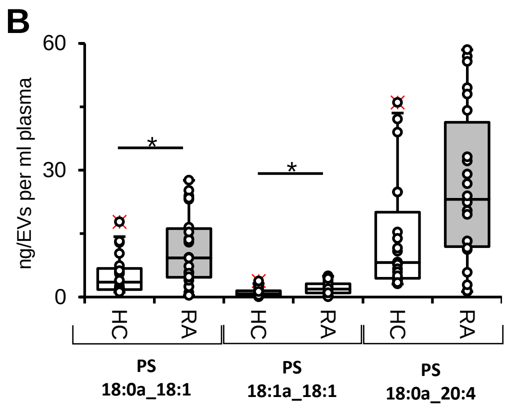

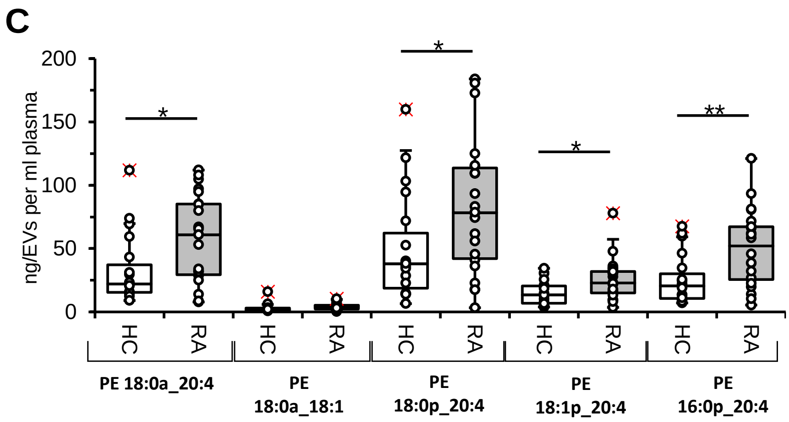

Supplementary Figure 1. **Extracellular vesicles from RA patients have increased levels of total PE and PS, but similar external facing PE.** *Panel A. PE externalization in EVs from RA patients and HC are similar.* EV particles were isolated followed by biotinylation of externalized lipids, as described in Methods. Lipids were extracted from plasma EVs isolated from HC (n = 19) and RA patients (n = 21), as described in Methods, and analyzed by LC/MS/MS. Data was analyzed using multiple Mann-Whitney test (*p<0.05, **p<0.001). *Panels, B,C. Total PS and PE are increased in EVs from RA patients.* Total PS and PE were analyzed by LC/MS/MS as described in Methods (ng/EVs per ml plasma). Lipids were extracted from plasma from HC (n = 19) and RA patients (n = 22). Data were analyzed using multiple Mann-Whitney test (*p<0.05, **p<0.001).

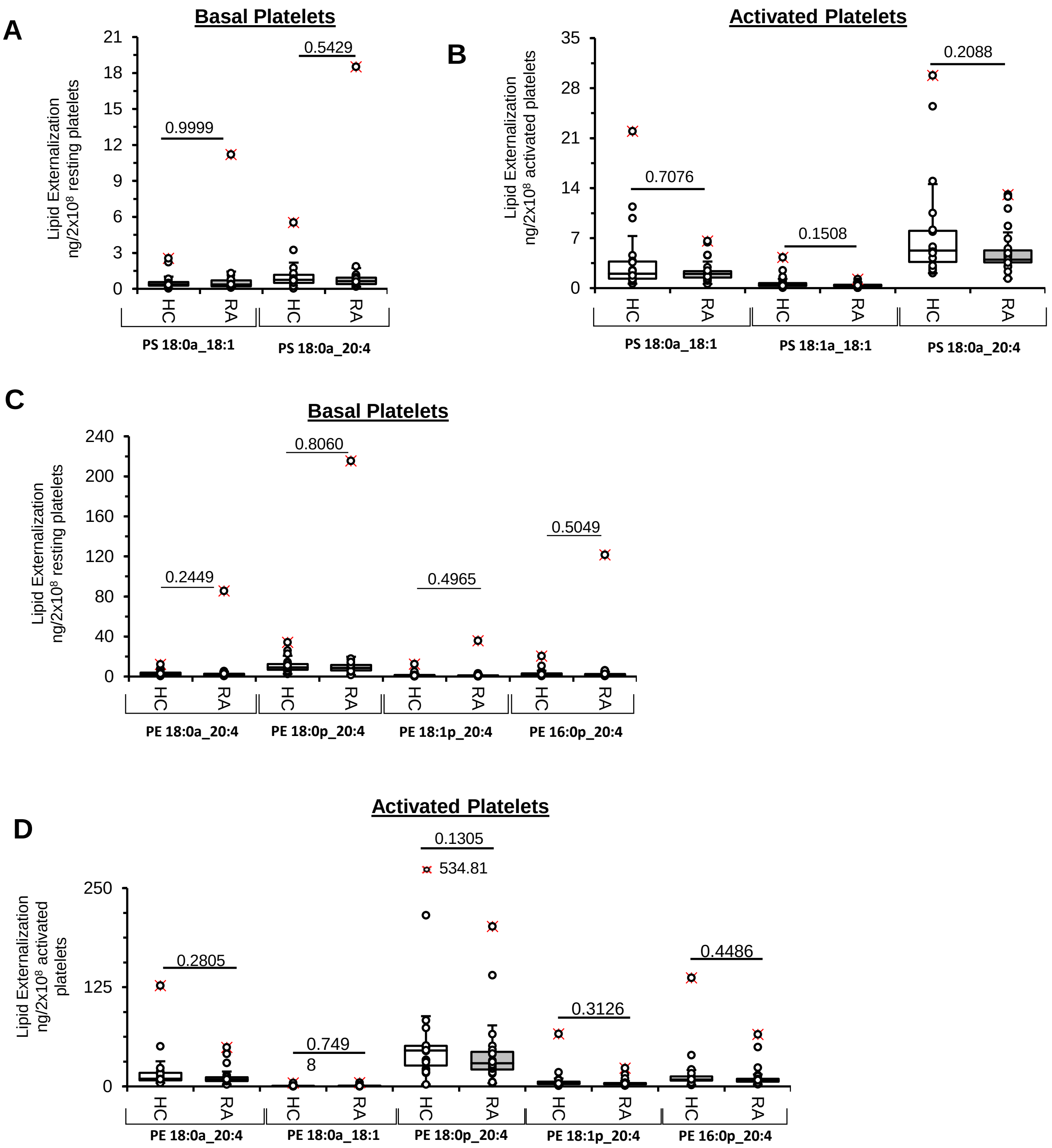
A
Basal Platelets
Lipid Externalization ng/2x10^8 resting platelets
0.9999
0.5429
HC
RA
PS 18:0a_18:1
PS 18:0a_20:4
B
Activated Platelets
Lipid Externalization ng/2x10^8 activated platelets
0.7076
0.1508
0.2088
PS 18:0a_18:1
PS 18:1a_18:1
PS 18:0a_20:4
C
Basal Platelets
Lipid Externalization ng/2x10^8 resting platelets
0.2449
0.8060
0.4965
0.5049
PE 18:0a_20:4
PE 18:0p_20:4
PE 18:1p_20:4
PE 16:0p_20:4
D
Activated Platelets
Lipid Externalization ng/2x10^8 activated platelets
0.2805
0.749
8
0.1305
534.81
0.3126
0.4486
PE 18:0a_20:4
PE 18:0a_18:1
PE 18:0p_20:4
PE 18:1p_20:4
PE 16:0p_20:4

Supplementary Figure 2. **Platelets from RA patients externalize similar aPLs to HC.** *Panel A. PS externalization in resting platelets is similar between RA patients and HC.* Externalized aPL were extracted from resting platelets, as described in Methods, from HC (n = 20) and RA patients (n = 21), and analyzed using LC/MS/MS and quantified aPL. *Panel B. Externalization of PS in platelets from RA patients and HC are not significantly different.* Platelets were isolated from HC (n = 20) and RA patients (n = 22) and activated using thrombin (0.2 U/mL). Externalized aPL were extracted and analyzed using LC/MS/MS. *Panel C. PE externalization in resting platelets is similar between RA patients and HC.* aPL were extracted from resting platelets, as described in Methods, from HC (n = 20) and RA patients (n = 21), and analyzed using LC/MS/MS. *Panel D. Externalization of PE in platelets from RA patients and HC are not significantly different.* Platelets were isolated from HC (n = 20) and RA patients (n = 22) and activated with thrombin (0.2 U/mL). Lipids were extracted and analyzed by LC/MS/MS. Data were analyzed using multiple Mann-Whitney tests.

Supplementary Figure 3

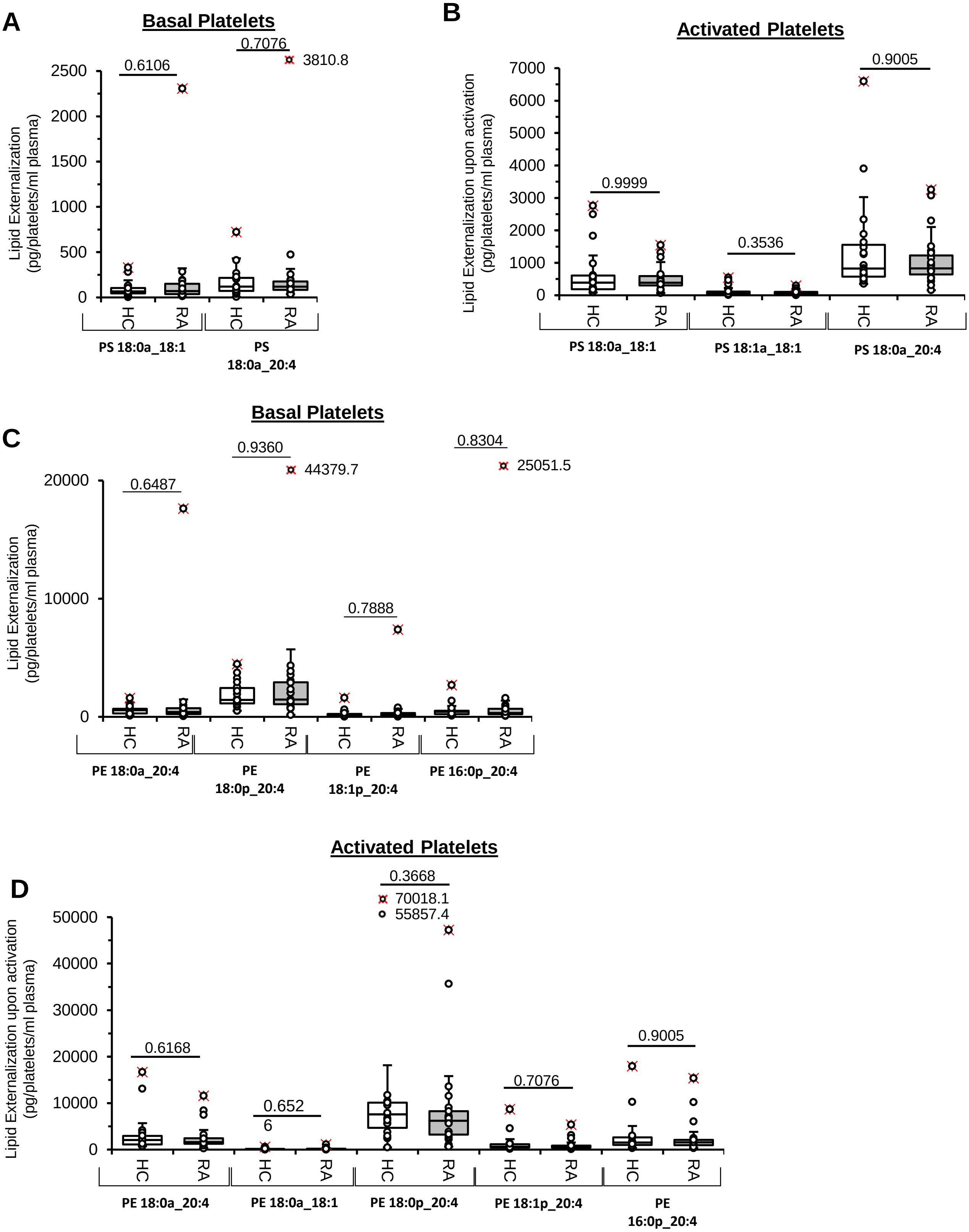

Supplementary Figure 3. **Normalizing data to platelet count doesn't increase overall impact of RA on externalized aPL levels**. *Panels A,B. Externalization of PS in platelets is similar between RA patients and HC following normalization to platelet counts.* aPL were extracted from resting or thrombin-activated platelets, from HC (n = 19 for both) or RA patients (n = 21 for resting and n = 23 for activated), and analyzed by LC/MS/MS. The amounts of individual externalized PS species, in resting and activated cells were determined and expressed as number of platelets isolated from 1 ml of plasma. *Panels C,D. Externalization of PE in platelets is similar between RA patients and HC following normalization to platelet counts.* aPL were extracted from resting or thrombin-activated platelets, from HC (n = 19 for both) or RA patients (n = 21 for resting and n = 23 for activated), and analyzed by LC/MS/MS. The amounts of individual externalized PS species, in resting and activated cells were determined and expressed as number of platelets isolated from 1 ml of plasma. Data were analyzed using multiple Mann-Whitney tests.

Supplementary Figure 4

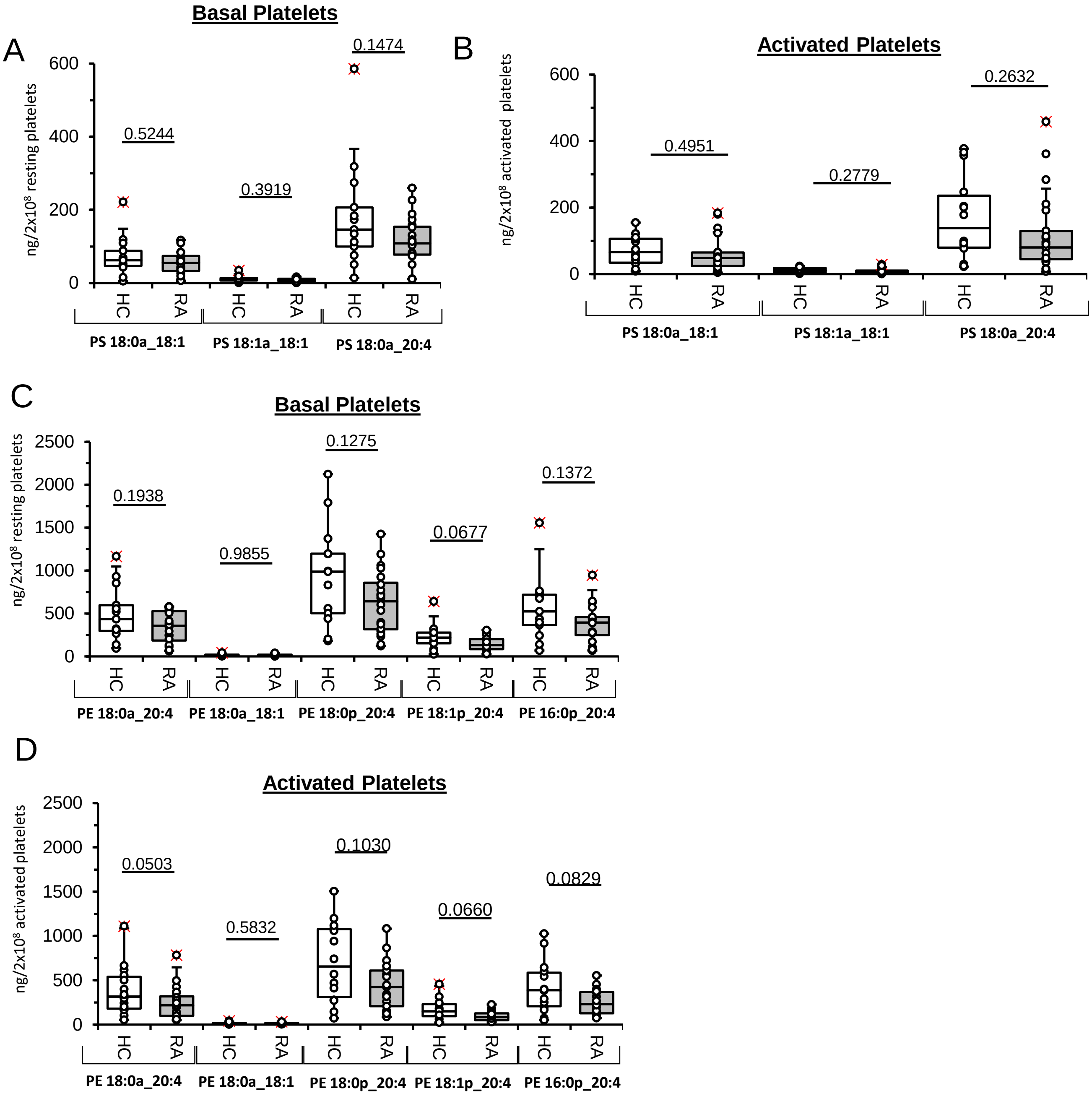

Supplementary Figure 4. **Total PS and PE levels in platelets are unchanged in RA.** *Panels, A,B. Total amount of PS in platelets, either resting or activated is similar between RA patients and HC.* Lipids were extracted from resting or thrombin-activated platelets from HC (n = 13 and n = 14, respectively) and RA patients (n = 20 and n = 21, respectively), before and after thrombin activation, as outlined in Methods, and analyzed using LC/MS/MS. Data are analyzed using multiple Mann-Whitney tests. *Panels C,D. Total amount of PE in activated platelets from RA patients is similar to HC.* Lipids from resting and activated platelets from RA patients (n = 13 and n = 14, respectively) and HC (n = 20 and n = 21, respectively) were extracted and analyzed by LC/MS/MS as described in Methods. Data were analyzed using multiple Mann-

Supplementary Figure 5

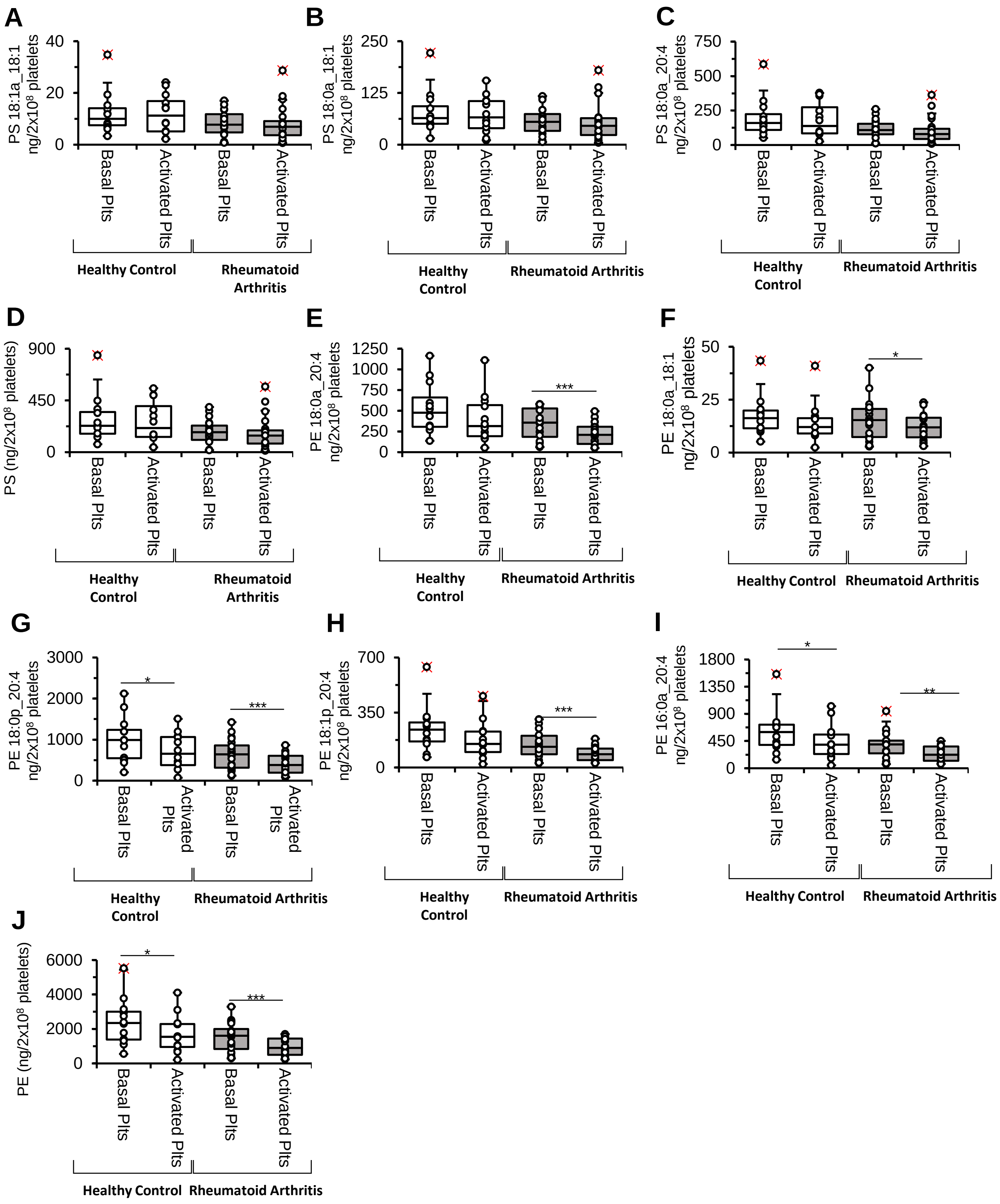

Supplementary Figure 5. **Total PE is decreased upon activation of platelets, especially in RA.** aPL were extracted from resting platelets, and following activation with thrombin, from HC (n = 12 for both) or RA patients (n = 20 for both) and analyzed by LC/MS/MS as described in Methods. *Panels A,D. Total PS is unchanged in platelets from either HC or RA patients after activation.* Total PS were measured in resting or activated platelets from HC or RA patients. *Panels E,J. Total PE is decreased in platelets from RA patients and HC after activation.* Total PE were measured in resting or activated platelets from HC and RA patients. Data were analyzed using paired Student T-test, comparing resting and stimulated samples, for HC and RA patients, separately. (*p<0.05, **p<0.01, *** p<0.001, ****p<0.0001).

Supplementary Figure 6

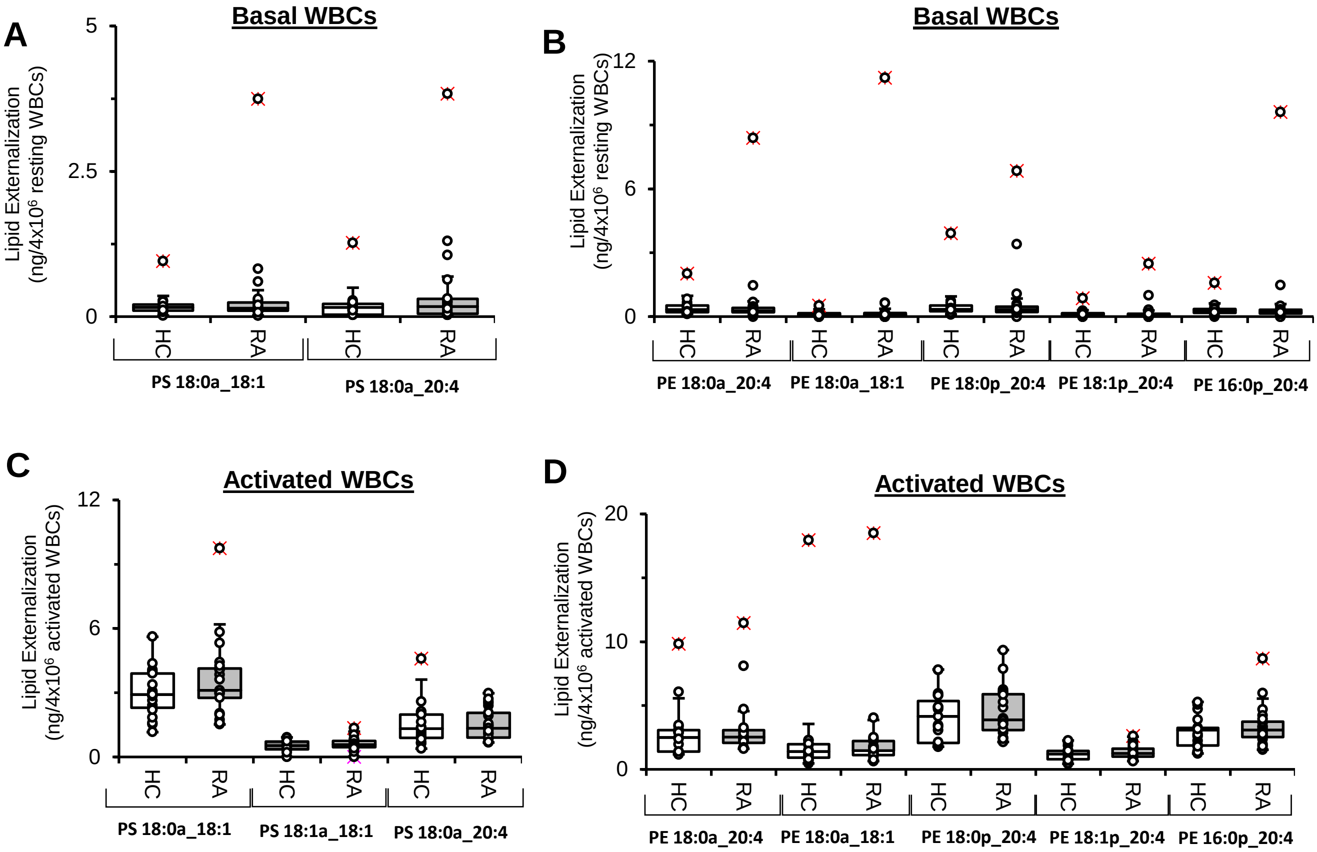

Supplementary Figure 6. **Externalized aPL in WBC are similar between RA and HC.** *Panels A,B. aPL externalization in resting WBC is similar for RA patients and HC.* aPL were extracted from resting WBC isolated from HC (n = 20) or RA patients (n = 23) and analyzed by LC/MS/MS. *Panels C,D. aPL externalization in activated WBC is similar between RA patients and HC.* aPL were also extracted from isolated WBC from HC (n = 19) or RA patients (n = 20) and following activation with Ca^{2+} ionophore (10 µM), as described in Methods, and analyzed by LC/MS/MS. Data were analyzed using multiple Mann-Whitney tests.

Supplementary Figure 7

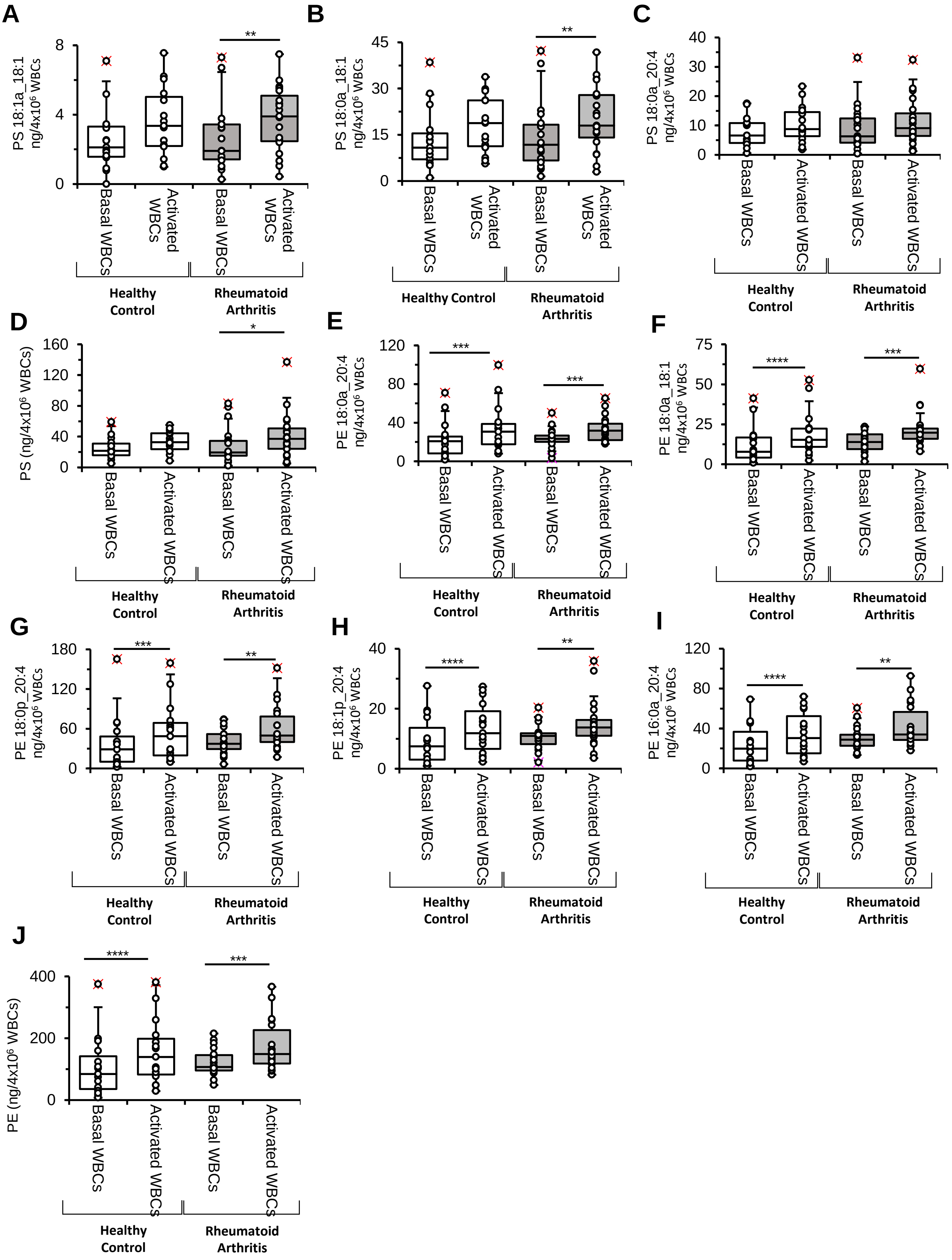

Supplementary Figure 7. **Total aPL increases on activation of WBC in either RA or HC.** aPL were extracted from resting WBCs and following activation with Ca^{2+} ionophore (10 µM), isolated from HC (n = 15 for both) or RA patients (n = 19 for both), and analyzed by LC/MS/MS, as described in Methods. *Panels A,D. Total PS is increased in WBC from RA patients after activation.* Total PS were measured in resting or activated WBC from HC or RA patients. *Panels E,J. Total PE is increased in WBC from RA patients and HC after activation.* Total PE were measured in resting or activated WBC from HC and RA patients. Data were analyzed using paired Student T-test, comparing resting and stimulated samples, for HC and RA patients, separately. (*p<0.05, **p<0.01, *** p<0.001, ****p<0.0001).

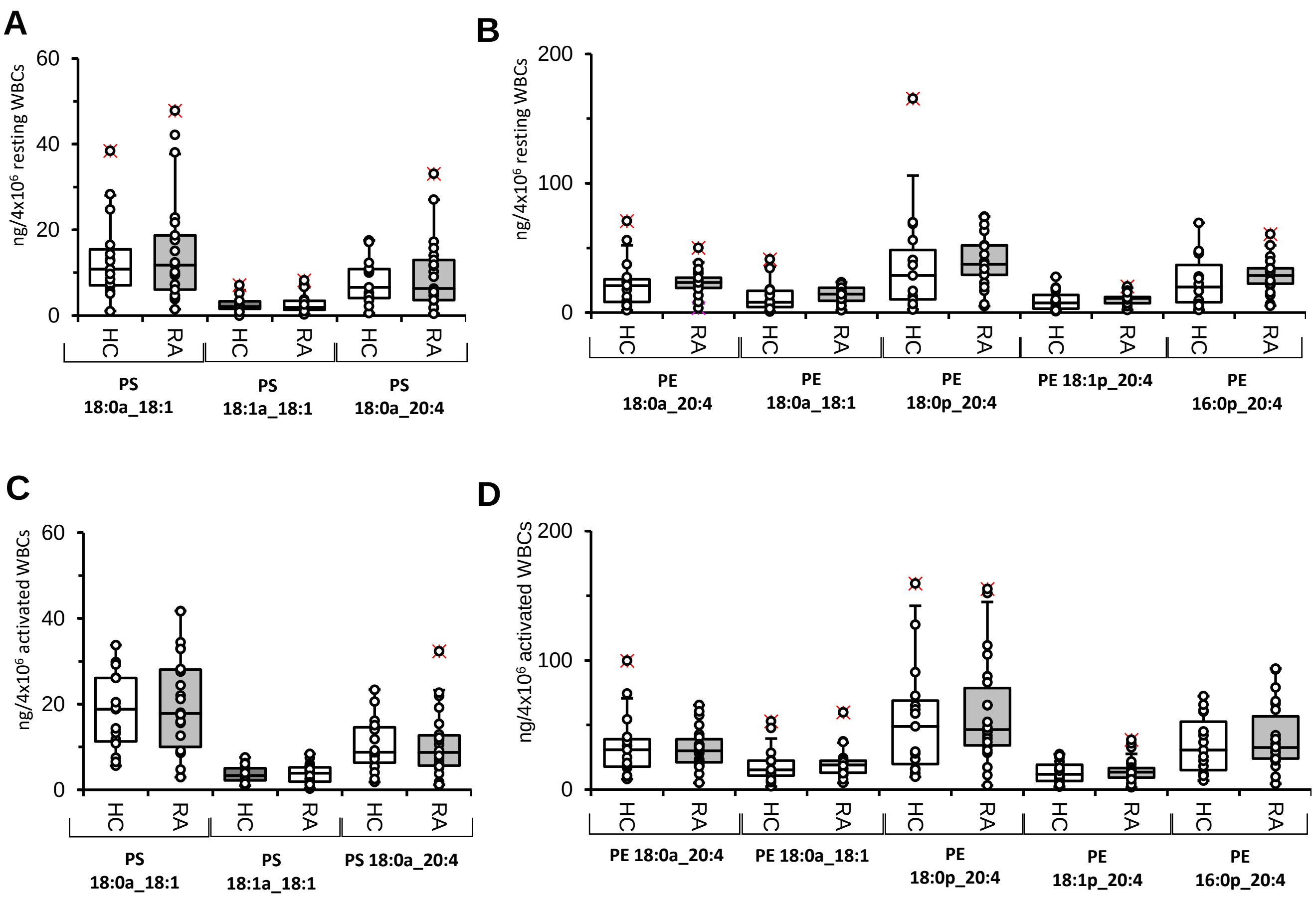

Supplementary Figure 8. WBC from RA patients have similar levels of PS and PE to HC. *Panels A,B. Total aPL in resting WBC is similar between RA patients and HC.* aPL were extracted from resting WBC isolated from HC (n = 15) or RA patients (n = 21), as described in Methods and analyzed by LC/MS/MS. *Panels C,D. Total aPL in activated WBC is similar between RA patients and HC.* Lipids were extracted from isolated WBC from HC (n = 15) and RA patients (n = 22), following activation with Ca^{2+} ionophore (10 μM), as described in Methods, and analyzed by LC/MS/MS. Data were analyzed using multiple Mann-Whitney tests.

Supplementary Figure 9

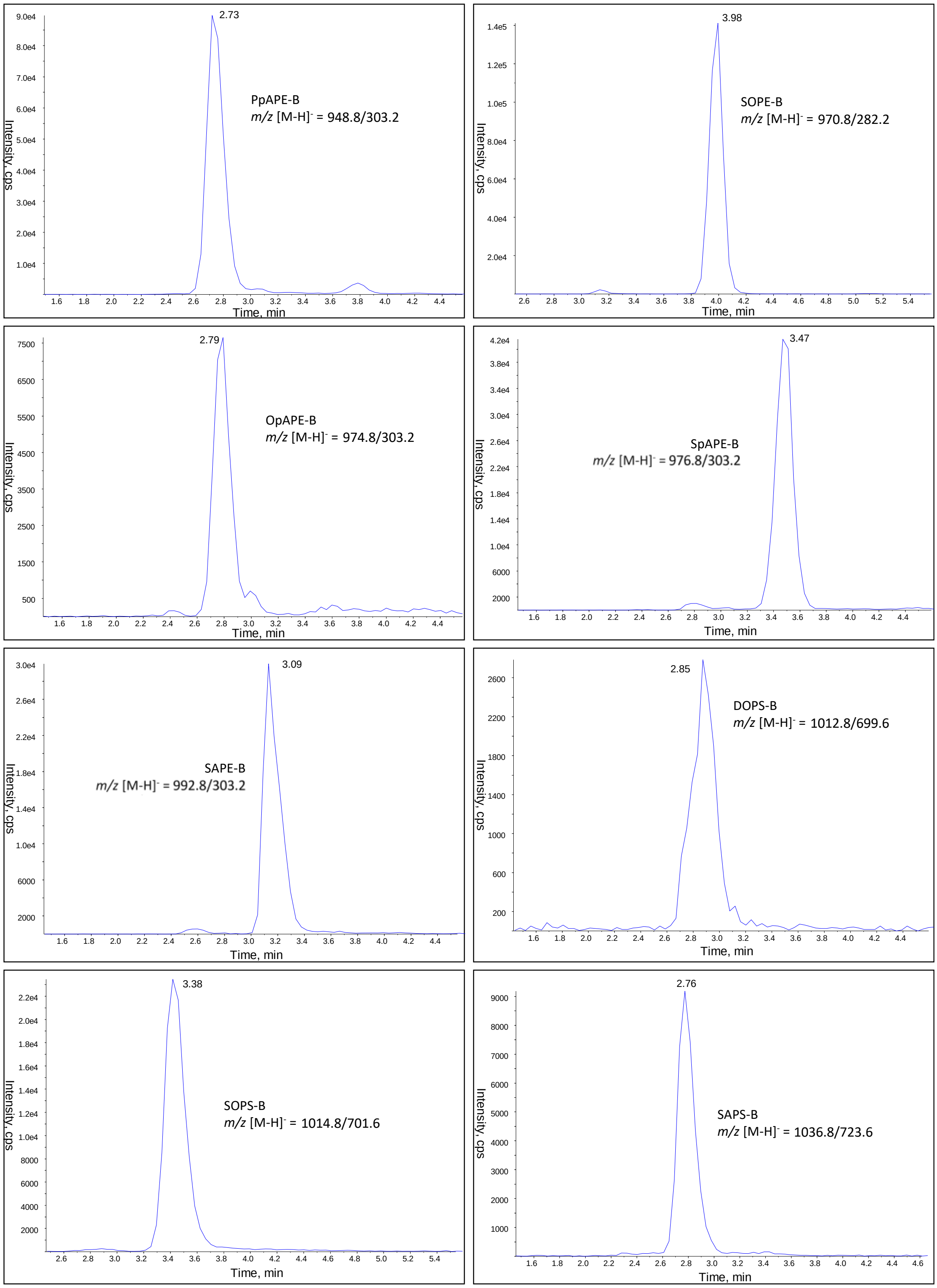

Supplementary Figure 9. Representative chromatograms of aPL LC/MS/MS analysis Lipid extracts were separated using reverse-phase LC/MS/MS, as described Methods. Screenshots were taken from Multiquant software.
